# The Development and Evolution of Substrate Materials for Brain–Computer Interfaces

**DOI:** 10.3390/polym18202569

**Published:** 2026-10-19

**Authors:** Jiujiang Ji, Yidan Gao, Juan Liu, Mei Tian, Yen Wei

**Affiliations:** 1 The Key Laboratory of Bioorganic Phosphorus Chemistry & Chemical Biology, Department of Chemistry, Tsinghua University, Beijing 100084, China; 2 State Key Laboratory of Genetic Engineering, School of Life Sciences, Human Phenome Institute, Zhangjiang Fudan International Innovation Center, Fudan University, Shanghai 201203, China; 3 School of Future Medicine, Beijing University of Chinese Medicine, Beijing 100102, China; 4 School of Materials Science and Engineering, North Minzu University, Yinchuan 750021, China; 5 Department of Chemistry and Center for Nanotechnology, Chung Yuan Christian University, Chung Li District, Taoyuan 32023, Taiwan

**Keywords:** Brain–Computer Interfaces (BCIs), implantable neural microelectrodes, biomechanical mismatch, electrospun nanofibers, chronic bio-integration

## Abstract

Brain–Computer Interfaces (BCIs) hold tremendous potential for neurological therapeutics and neuroprosthetics; however, their long-term clinical viability is fundamentally limited by chronic interfacial failure. This failure originates from the profound biomechanical mismatch between synthetic microelectrodes and ultra-soft brain tissue, which inevitably triggers persistent mechanical trauma, severe foreign body response (FBR), and insulating glial scarring. In this review, we systematically trace the material and structural evolution of implantable neural interfaces designed to bridge this critical gap. We detail the chronological paradigm shift from rigid inorganic substrates (e.g., silicon) to flexible yet inextensible polymers (e.g., Polyimide, SU-8) for conformal mapping, and subsequently to intrinsically stretchable elastomers (e.g., polydimethylsiloxane polyurethane) that effectively absorb dynamic physiological micro-shear forces. Most importantly, we highlight the emerging frontier of electrospun nanofibrous microelectrodes. By uniquely combining macroscopic tissue-level stretchability with an interconnected, highly permeable 3D topography that mimics the native extracellular matrix (ECM), these biomimetic scaffolds successfully overcome the metabolic limitations of dense elastomeric films. Finally, we discuss current challenges and future perspectives, ranging from scalable high-density manufacturing and active biochemical modulation to fully untethered integration, charting a comprehensive roadmap for next-generation, chronically stable neural interfaces.

## 1. Introduction

**Figure 1:**
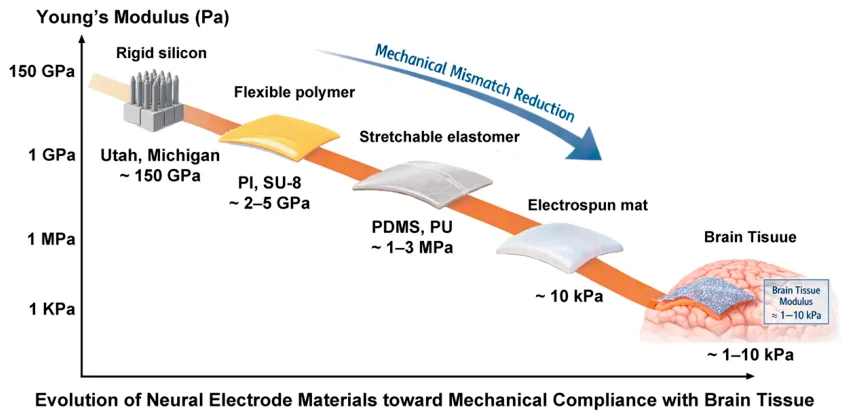
Evolution of neural electrode materials aimed at minimizing the mechanical mismatch with brain tissue.

Brain–Computer Interfaces (BCIs), serving as a direct bridge connecting the central nervous system to external electronic devices, have demonstrated tremendous transformative potential in fundamental neurophysiological research, neuropsychiatric disorder therapies, and neuroprosthetic rehabilitation [1,2]. Bridging the physical gap between artificial implants and soft biological systems has driven a paradigm shift in neural interfaces toward tissue-level mechanical compliance (Figure 1). Achieving long-term, stable extraction of high-fidelity electrophysiological signals with high spatial resolution is the core prerequisite for advancing the clinical translation of BCI technology [3,4]. However, a profound mechanical mismatch spanning several orders of magnitude exists between traditional silicon-based or metallic implantable neural microelectrodes (Young’s modulus of ~100–200 GPa) and soft, fragile brain tissue (~1–10 kPa) [5,6]. Driven by physiological micromotions such as cardiac pulsations and respiration, this rigid contact generates continuous shear stress at the tissue–electrode interface [7,8]. This mechanical trauma leads to irreversible neuronal damage and inevitably triggers a severe foreign body response (FBR) [9,10]. The subsequent chronic neuroinflammation and dense glial scar formation not only significantly increase interfacial impedance but also physically isolate the electrodes from target neurons, ultimately resulting in signal attenuation or complete failure during chronic implantation [11,12].

To mitigate this chronic interfacial failure, the structural and material design of neural electrodes has undergone a profound evolution. Historically, the pursuit of bridging this mechanical mismatch has driven a paradigm shift from rigid inorganic substrates to advanced functional polymers [13,14]. Early classic neural microelectrode arrays (e.g., Utah and Michigan arrays) relied extensively on rigid silicon. Although silicon offers outstanding microfabrication precision and semiconductor properties, its extreme rigidity (~150 GPa) causes the electrodes to act as “micro-blades” under physiological micromotions, slicing through the ultra-soft tissue and exacerbating glial scarring [15,16]. To alleviate this mechanical trauma, flexible yet inextensible polymers such as polyimide (PI), Parylene C, and SU-8 were introduced [17,18]. PI, favored for its excellent chemical stability, insulation, and seamless compatibility with standard microelectromechanical systems (MEMS) processing, served as the cornerstone for the first generation of flexible electrodes [19,20]. Fundamentally, the selection of PI as a premier neural interface substrate is deeply rooted in its chemical architecture. Characterized by a highly stable aromatic imide ring backbone, PI exhibits exceptional chemical inertness, resistance to enzymatic degradation, and outstanding dielectric properties. These intrinsic chemical traits ensure that the polymer remains highly insulating and stable, effectively preventing ionic leakage and signal crosstalk in the harsh, corrosive physiological environment of the brain. However, with moduli remaining in the low gigapascal range (e.g., ~2.5 GPa for PI), these polymers are bendable but lack in-plane stretchability. This limitation restricts their ability to fully conform to the complex 3D sulci and dynamic deformations of the cerebral cortex [21,22].

Seeking to further close the mechanical gap, stretchable elastomers like polydimethylsiloxane (PDMS) and polyurethane (PU) emerged as research hotspots [23,24]. With a modulus (~1–3 megapascal (MPa)) significantly closer to that of soft neural tissues, PDMS effectively absorbs interfacial micro-shear forces [25,26]. Nevertheless, traditional elastomeric films present inherent biological limitations: their strong hydrophobicity facilitates non-specific protein adsorption, while their dense, impermeable structure lacks the biomimetic topographical cues necessary to emulate the natural extracellular matrix (ECM) [27,28]. To overcome this long-standing bottleneck, flexible bioelectronics based on functional polymers and nanomaterials have achieved leapfrog development. Compared to traditional devices, flexible bio-interfacial materials such as electrospun nanofibers can significantly alleviate mechanical stress at the tissue interface due to their excellent mechanical compliance and biomimetic topological structures [29,30]. As the field has rapidly expanded, the application scenarios for these ultra-flexible bioelectronic electrodes have transcended localized cortical recordings. They now seamlessly conform to a wide array of complex, highly dynamic biological surfaces to enable comprehensive multimodal physiological monitoring [31,32]. This highly conformal design establishes a tight and stable electrical coupling across various anatomical environments while maximally eliminating mechanical compression and trauma to soft tissues, paving the way for chronic, highly stable biological integration [33,34].

In this review article, we trace the material and structural evolution of implantable neural microelectrodes, driven by the critical imperative to bridge the biomechanical gap between electronic devices and soft brain tissue [35,36]. We begin by examining the historical foundations and inherent chronic limitations of traditional rigid inorganic substrates, such as silicon-based arrays. The discussion then transitions to flexible polymeric microelectrodes, highlighting how bendable materials like PI and SU-8 have enabled conformal surface mapping and multimodal integration, despite their lack of in-plane stretchability. Next, we explore the paradigm shift toward stretchable elastomeric microelectrodes, detailing how highly compliant networks like PDMS and PU effectively absorb dynamic physiological micromotions. Finally, we spotlight the emerging frontier of electrospun nanofibrous microelectrodes. By uniquely combining macroscopic stretchability with microscopic interconnected porosity and an ECM-mimicking topology, we illustrate how these biomimetic scaffolds overcome the critical permeability deficits of solid elastomers. Through this chronological analysis, we aim to provide a clear roadmap of bio-interface design strategies, ultimately inspiring researchers to further explore the clinical utility and translational potential of electrospun flexible substrates for next-generation, chronically stable brain–computer interfaces. To provide a comprehensive overview of this material evolution, Table 1 summarizes the progressive stages of neural microelectrodes, comparing their distinct mechanical properties, structural advantages, and clinical limitations.

## 2. Traditional Rigid Inorganic Microelectrodes

**Figure 2:**
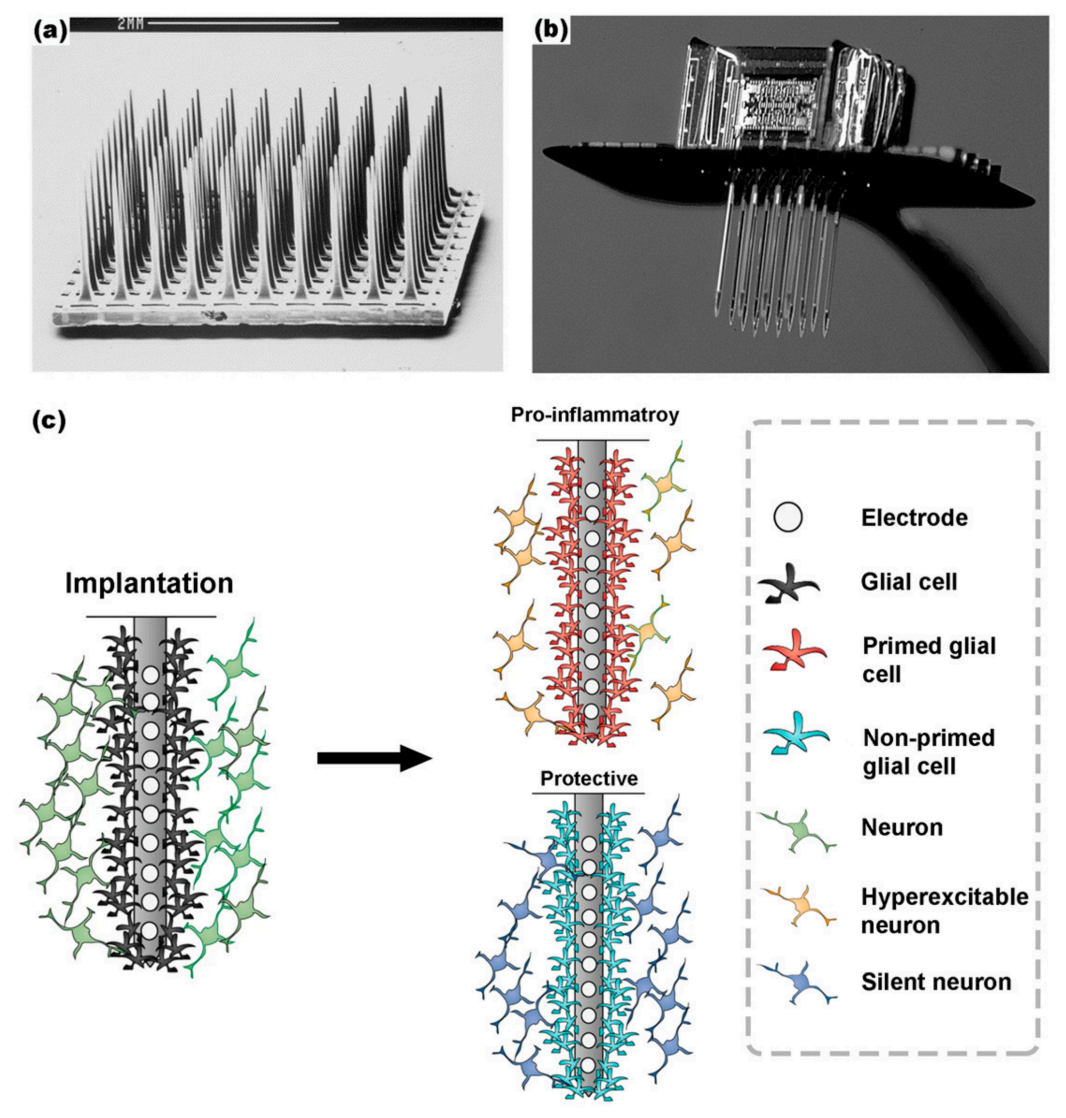
(**a**) Scanning electron micrograph of a Utah array [37]. (**b**) Photograph of an active Michigan [38]. (**c**) Schematic illustrating the mechanisms of glial state transition and the immune rejection process following neural electrode implantation [39].

In the nascent stages of BCI technology, rigid inorganic microelectrode arrays fabricated via traditional MEMS techniques dominated the field [52,53]. The most representative architectures from this era are the classic Utah arrays [37] and Michigan arrays [38] (Figure 2a,b). Relying primarily on rigid silicon substrates, these electrodes exhibit a high Young’s modulus of approximately 150 GPa [54]. This intrinsic mechanical rigidity and sharp geometry endow the microelectrodes with the capability to easily pierce the pia mater and achieve precise cortical implantation without the assistance of auxiliary tools. Furthermore, their mature semiconductor properties offer inherent advantages for the high-density integration of recording sites and the acquisition of high-fidelity electrophysiological signals during the acute phase [55,56].

However, this extreme material rigidity has emerged as the primary bottleneck restricting their viability for long-term chronic implantation [57]. Ultra-soft natural brain tissue possesses a Young’s modulus of merely 1–10 kPa, resulting in a profound mechanical mismatch spanning several orders of magnitude between the rigid implant and the surrounding neural environment [58,59]. Under the constant influence of physiological micromotions driven by respiration and cardiac pulsations, these 150 GPa rigid silicon arrays act as “micro-blades” embedded within the soft brain. This rigid contact generates relentless shear stress and micro-displacements at the tissue–electrode interface, inflicting chronic mechanical micro-trauma [60,61].

Inevitably, this sustained mechanical insult triggers a severe FBR, leading to the apoptosis and degeneration of adjacent neurons while stimulating the extensive recruitment and activation of microglia and astrocytes around the electrode [39,62] (Figure 2c). Ultimately, this inflammatory cascade culminates in the encapsulation of the device by a dense, insulating glial scar. Beyond biological rejection, these bare inorganic microelectrodes also suffer from inherent electrochemical limitations. As the geometric area of recording sites is minimized to achieve single-neuron resolution, the interfacial electrical impedance increases drastically while the charge injection capacity remains insufficient for effective long-term stimulation [63,64]. The combination of structural isolation by the glial scar and suboptimal electrochemical properties leads to a progressive attenuation of the signal-to-noise ratio, eventually resulting in complete device failure [65]. To overcome this fatal interfacial mechanical discrepancy, the evolution of neural electrode materials has undergone a profound paradigm shift, pivoting toward advanced materials designed to achieve seamless mechanical compliance with brain tissue [66].

## 3. Flexible Polymeric Microelectrodes

Although early flexible yet inextensible polymers (such as PI and Parylene C) significantly reduced the structural stiffness of neural probes compared to rigid inorganic silicon, their Young’s moduli (in the low gigapascal range) remain several orders of magnitude higher than that of ultra-soft cortical tissue (~1–10 kPa) [67,68]. Furthermore, a major clinical bottleneck for flexible polymeric neural probes is the difficulty of implantation, as they are highly susceptible to buckling failure during tissue insertion [69,70].

**Figure 3:**
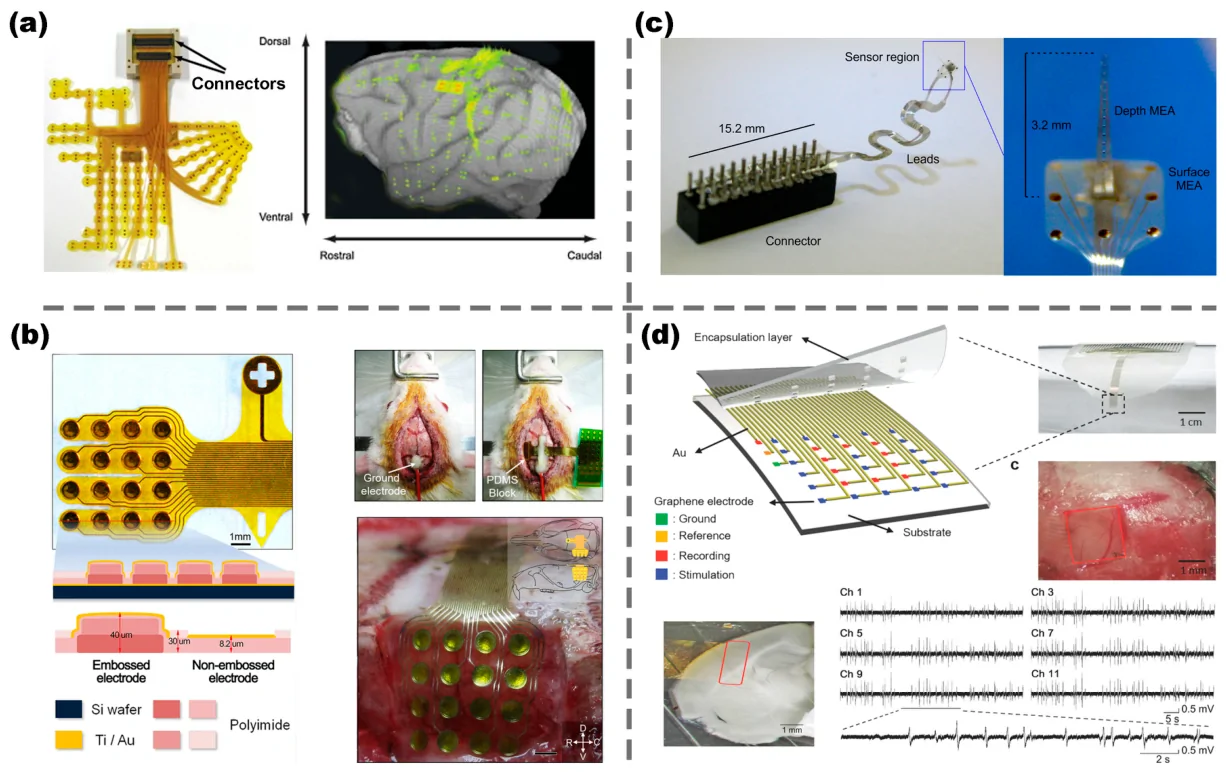
(**a**) Photograph of the assembled ECoG electrode array and connectors, and the mapped locations of the electrodes on a 3D-rendered image of the brain [40]. (**b**) Optical images and cross-sectional schematic of the electrode array design, along with photographs of the device implanted on the exposed auditory cortex [41]. (**c**) Optical photograph of the integrated device assembly and a magnified view of the sensor region featuring the MEA [42]. (**d**) Schematic illustration of a graphene-based seizure sensor, and representative interictal-like activities recorded in vivo using the multichannel graphene electrode [43].

Despite these challenges in penetrating applications, the excellent out-of-plane bendability and superior photolithographic compatibility of these polymers make them ideal candidates for non-penetrating, surface-conformal neural interfaces [71,72]. For instance, as shown in Figure 3a, Fukushima et al. [40] developed a novel compartmental electrocorticography (ECoG) array for macaques using a flexible PI substrate. Featuring a PI insulating film sandwiched between copper layers and gold-plated contacts, the device integrated 256 electrodes, each 0.8 mm in diameter, to form 128 bipolar sites with a 1.4 mm inter-electrode spacing. This fabrication process achieved an ultra-thin total device thickness of just 53–56 µm, maintaining low mean impedances ranging from 140 kΩ at 10 Hz to 0.9 kΩ at 10 kHz. Benefiting from PI’s flexibility, this compartmental design enabled simultaneous conformal coverage across complex cortical surfaces, including the deeply buried supratemporal plane. In awake macaques, it successfully captured robust sensory-evoked potentials and high-gamma responses, accurately recording auditory response latencies as short as ~10 ms and visual response peaks at around 120 ms. Notably, these signal recording capabilities remained highly stable for 5 and 6 months post-implantation, respectively. Furthermore, while standard monopolar recordings were highly susceptible to motion interference, where chewing hard foods produced an ~8-fold greater peak artifact than soft foods, the array’s bipolar configuration effectively eliminated these chewing artifacts to ensure high-fidelity signal extraction.

Building upon the success of subdural conformal arrays, researchers have further leveraged PI’s mechanical compliance to push the boundaries toward even less invasive epicranial interfaces [41]. To further illustrate the versatility of PI in minimally invasive neural interfaces, Kim et al. [73] developed an electroplating-free, 16-channel PI microelectrode array designed specifically for epicranial recordings (Figure 3b). Leveraging PI’s inherent flexibility, the device featured an “embossed” recording site structure (1 mm diameter, 0.5 mm spacing) that maximized conformal contact with the curved rat skull, thereby enhancing contact impedance without relying on fragile platinum-black electroplating. Furthermore, the integration of ground lines between signal channels within the PI substrate successfully reduced inter-channel crosstalk by approximately 15 dB. During in vivo auditory evoked potential (AEP) mapping in rats, this flexible array extracted high-quality cortical signals directly from the skull surface, accurately capturing characteristic AEP peaks including N9 (~8.9 ms), P22 (~21.6 ms), and N39 (~39.1 ms). It also demonstrated lower signal variability (68.8 ± 3.5 μV compared to 86.6 ± 9.3 μV for non-embossed designs), highlighting its efficacy for stable, low-noise, and minimally invasive neural monitoring.

While PI-based surface arrays excel in capturing broad cortical activity, unraveling complex neural circuits often necessitates the simultaneous mapping of both surface potentials and deep-tissue laminar signals. This demand has driven the development of multimodal hybrid probes, where SU-8, a polymer with excellent high-aspect-ratio photopatternability, is integrated to provide necessary structural integrity for tissue penetration. To advance multimodal integration, Márton et al. [42] developed a fully flexible 3D microelectrode array (MEA) using an SU-8 top insulator and a PI base. This device uniquely combined an 8-channel surface ECoG array featuring 200 μm diameter sites with a 16-channel penetrating shank containing 30 μm diameter sites, enabling simultaneous recording of surface local field potentials and intracortical laminar signals (Figure 3c). To optimize signal extraction, the depth electrodes were electrochemically platinized, which drastically reduced their 1 kHz impedance magnitude from 559.5 ± 148.4 kΩ down to 27.6 ± 8 kΩ. In acute rat experiments, it successfully captured 1–1.5 Hz slow-wave oscillations and high-quality current source density profiles. Notably, during a 15-week chronic implantation, the MEA consistently detected single-neuron action potentials. Throughout this extended period, the device maintained a stable single-unit yield of 4 to 7 per array, typical spike amplitudes of 30 to 50 μV, and average signal-to-noise amplitude ratios (SNAR) ranging from 2.42 to 4.69. These quantitative metrics decisively demonstrate the remarkable long-term reliability and in vivo stability of SU-8-encapsulated flexible probes for chronic neural monitoring.

Beyond passive signal recording, the next frontier in flexible bioelectronics lies in active electrotherapeutic interventions, which demand materials with extraordinary charge transfer capacities and extreme electrochemical durability. To quantitatively demonstrate the potential of SU-8 in advanced therapeutic interfaces, Park et al. [43] developed an ultrathin epidural array utilizing a 5 μm thick flexible SU-8 substrate to encapsulate chemically doped four-layer graphene electrodes (Figure 3d). The integration of multi-layer graphene on the SU-8 polymer significantly enhanced the electrochemical performance, achieving a high specific capacitance of 326.7 F/g. Mechanistically, this remarkable enhancement is driven by a combination of high-surface-area structural design and chemical modification. The multi-layer graphene architecture provides an extensive electroactive surface area that maximizes electrical double-layer capacitance (EDLC) at the bio-interface. Concurrently, chemical doping of the graphene layers drastically increases the charge carrier density and lowers sheet resistance, thereby facilitating highly efficient, rapid charge injection. Furthermore, the ultrathin SU-8 base offers a mechanically robust yet compliant scaffold that prevents the delamination of graphene under dynamic physiological stress, ensuring stable electrochemical transduction. Furthermore, the device exhibited remarkable durability, showing merely a ~5% impedance variation even after 90,000 continuous electrical stimulation cycles in artificial cerebrospinal fluid. During in vivo testing on epileptic mouse models, the conformal SU-8/graphene array delivered targeted sinusoidal high-frequency stimulation. This precise electrotherapy selectively suppressed ictal-like epileptic bursting, significantly reducing the mean spike counts from 66.64 to 44.91 and burst frequency from 1.68% to 0.94%, while leaving normal interictal signal transduction fully intact.

While graphene demonstrates exceptional electrochemical properties, the rapid advancement of nanomaterials has introduced several highly promising alternative candidates for flexible neural interfaces. For instance, Carbon Nanotubes (CNTs) offer comparable electrical conductivity with superior intrinsic mechanical entanglement, making them highly resilient against micro-cracking during tissue deformation. Alternatively, emerging 2D transition metal carbides/nitrides (MXenes) provide superior volumetric capacitance and intrinsic hydrophilicity, which significantly enhances charge transfer kinetics in aqueous physiological environments without the need for complex chemical doping. Furthermore, conductive polymers such as Poly(3,4-ethylenedioxythiophene) polystyrene sulfonate (PEDOT:PSS) remain a gold standard alternative due to their mixed ionic-electronic conductivity, effectively bridging the impedance gap between hard electrodes and soft tissues [74,75]. Exploring and integrating these alternative nanomaterials with flexible substrates like SU-8 and PI remains a critical pathway for customizing the electrochemical and mechanical profiles of next-generation BCI devices.

Despite their mechanical advantages over silicon, these synthetic polymers still present specific biocompatibility challenges. From a toxicological perspective, materials like SU-8 require rigorous post-processing. If not fully cross-linked and extensively purified, SU-8 can leach unreacted monomers, residual solvents, or antimony-based photoacid generators into the surrounding tissue, potentially inducing localized cytotoxicity and acute neuroinflammation. Furthermore, their foreign chemical surface inevitably provokes an immunological cascade, albeit milder than rigid silicon, which still leads to gradual fibrous encapsulation over chronic implantation periods.

In summary, the integration of flexible polymers such as PI and SU-8 has undeniably revolutionized neural interface technology. Their excellent out-of-plane bendability, high electrochemical stability, and seamless compatibility with standard microfabrication techniques have enabled the creation of sophisticated, high-density arrays capable of conformal epicranial mapping, deep-tissue laminar recording, and active electrotherapy [76]. However, a fundamental biomechanical limitation remains. With Young’s moduli lingering in the gigapascal range, these macromolecular networks are intrinsically inextensible. They completely lack the crucial in-plane stretchability required to dynamically accommodate the volumetric pulsations, complex 3D sulci, and continuous micro-deformations of the living brain, which inevitably leads to localized stress concentrations and chronic tissue friction over time [77]. To bridge this persistent mechanical gap and achieve true biomechanical synergy with soft neural tissues, the structural paradigm of neural electrodes was compelled to evolve further, ushering in the era of intrinsically stretchable elastomers such as PDMS and PU.

## 4. Stretchable Elastomeric Microelectrodes

Driven by the critical need to overcome the in-plane inextensibility and residual mechanical mismatch of early flexible substrates, intrinsically stretchable elastomers have emerged as a pivotal class of materials for next-generation neural interfaces. Elastomeric polymers, most notably PDMS and thermoplastic PU, exhibit Young’s moduli in the MPa range (typically 1–3 MPa), bringing them significantly closer to the viscoelastic properties of soft neural tissues [78,79]. Beyond their mechanical compliance, the widespread dominance of these elastomers in biomedical applications is fundamentally attributed to their chemical nature. PDMS is built upon a heavily cross-linked siloxane (Si-O-Si) backbone, which renders the material chemically inert, generally non-toxic, and highly biocompatible for the vast majority of the population. Similarly, PU is characterized by its versatile urethane linkages, which can be chemically tuned to balance structural stability with excellent baseline bio-integration, maintaining a chemically inert profile that minimizes acute tissue toxicity prior to any long-term degradation. Unlike their merely bendable predecessors, these highly elastic macromolecular networks can undergo substantial elongation and multiaxial deformation without structural failure. This unique mechanical compliance enables elastomeric bioelectrodes to intimately conform to the intricate, continuously pulsating topography of the cerebral cortex [80,81]. By effectively buffering the persistent micro-shear forces generated by respiratory and cardiac rhythms, PDMS and PU substrates drastically minimize acute mechanical trauma and substantially improve the chronic stability of BCIs.

**Figure 4:**
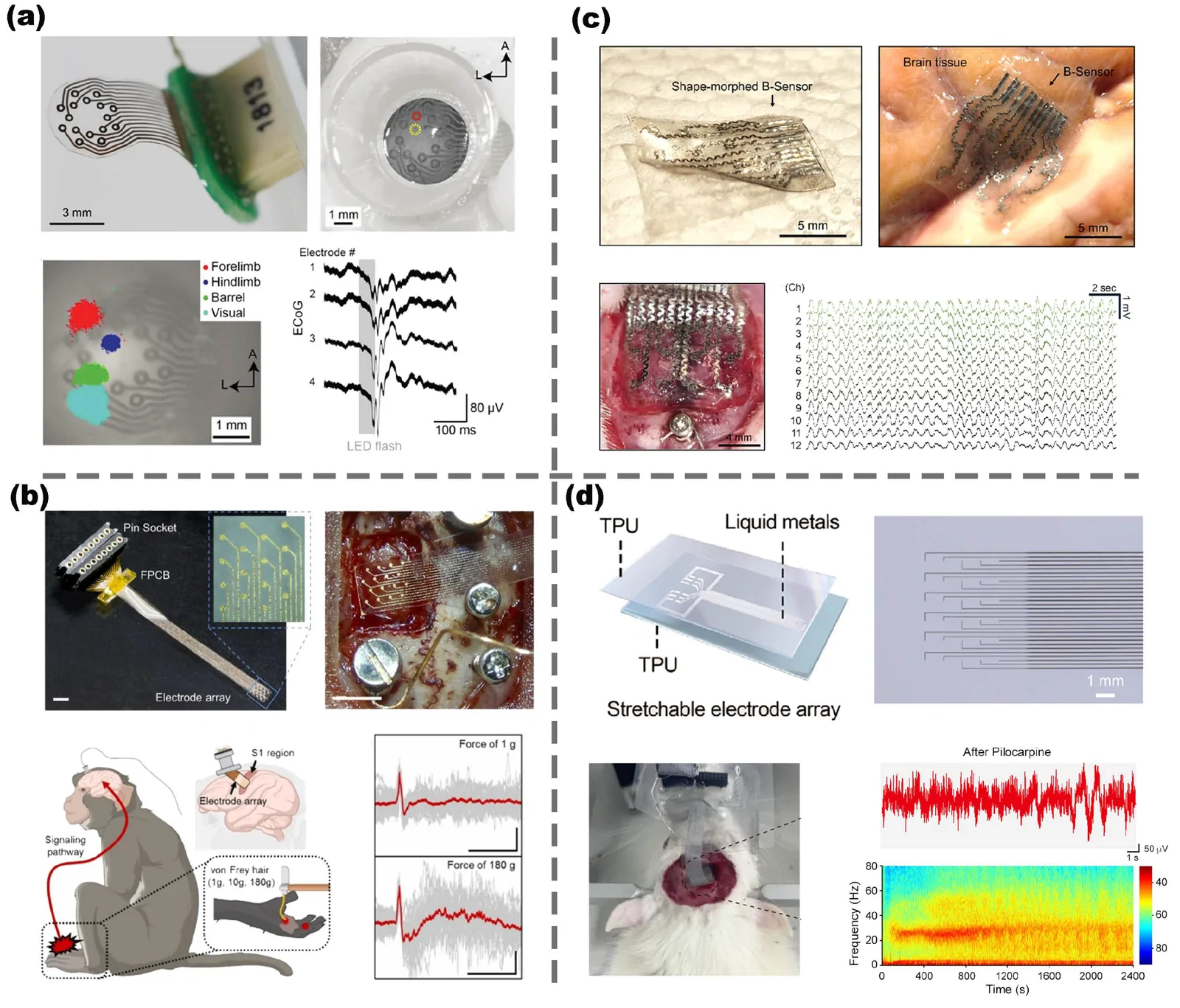
(**a**) Photographs of the ECoG array prior to imaging well attachment and after implantation over the dorsal cortex of a mouse. Modality-specific cortical regions for four sensory stimuli are overlaid on the wide-field image through the Opto-e-Dura. The colored regions correspond to the boundaries of peak activity derived from calcium activation maps [44]. (**b**) Photographs of the ECoG device before and after implantation. Also shown is a schematic illustration of chronic SEPs recording, alongside SEPs recorded under varying palm pressure levels (scale bars: 0.2 s, 100 μV) [45]. (**c**) Photographs demonstrating the shape-morphed, conformal contact of the B-Sensor and its implantation on the cortical surface, followed by baseline neural signals recorded under ketamine anesthesia [46]. (**d**) Schematic and structural photograph of a 30-channel stretchable microfluidic electrode array. An intraoperative image of the device implanted on a rat brain is shown alongside representative raw LFP data (top) and a time-frequency spectrogram of neural activity (bottom) during an epileptic seizure [47].

Among these elastomeric materials, PDMS has been extensively explored to leverage its optical clarity and tissue-like elasticity. As shown in Figure 4a, Renz et al. [44] utilized PDMS as a flexible implantable substrate to develop “Opto-e-Dura”, a stretchable 16-channel ECoG array. Leveraging the >90% optical transmittance and high elasticity (Young’s modulus of 724–881 kPa) of PDMS, the device enables simultaneous multimodal monitoring. It exhibits excellent chronic stability and biocompatibility, with no significant microglial immune response after 4–6 weeks of in vivo implantation. With porous platinum electrodes, the array achieves an extremely low impedance of 998 ± 93 Ω at 1 kHz (24 Ω cm^2^ at 1 Hz). Furthermore, the soft PDMS layer allows the array to be pierced by MEA without damage. This design successfully captured surface ECoG signals while synchronously detecting deep-tissue local field potentials (LFPs) and single-unit activity.

To further enhance the electrochemical stability and chronic performance of PDMS-based interfaces, structural and surface modifications are often employed. Building on this approach, Moon et al. [45] utilized parylene-treated PDMS as a soft, implantable substrate to fabricate long-term ECoG electrode arrays (Figure 4b). By employing 80 μm-thick parylene-deposited and parylene-filled PDMS, the flexible bio-interface achieved a low Young’s modulus of 18.1–28.7 MPa, ensuring highly conformal contact with the cortical surface. The parylene-filled PDMS electrodes demonstrated superior electrochemical performance with an initial impedance of 27.8 ± 11.2 kΩ at 1 kHz, which remained remarkably stable (55.7 ± 8.5 kΩ) even after 150 days of accelerated aging. In vivo, the device provided stable chronic recordings of somatosensory evoked potentials (SEPs) in non-human primates for up to 12 weeks. The array effectively detected high signal-to-noise ratio (SNR) electrophysiological signals in response to variable mechanical stimuli (1–300 g), allowing for precise spatial mapping of distinct sensory cortical areas.

Parallel to the advancements in PDMS, PU and its derivatives have emerged as highly adaptable alternatives, offering unique characteristics such as tunable biodegradability and dynamic stress-relaxation. Demonstrating this versatility, Choi et al. [46] utilized a polycarbonate-based polyurethane (PPU) as an implantable flexible substrate to develop a shape-morphable and fully biodegradable brain-adhesive sensor (Figure 4c). Featuring a Young’s modulus of 1.4 MPa and rapid stress relaxation (dissipating 96% of initial stress within 60 s), the polyurethane substrate conforms closely to the dynamic cerebral cortex and achieves a robust tissue adhesive strength of 4.4 kPa. The device provides artifact-free magnetic resonance imaging (MRI) compatibility and degrades naturally in approximately six months under physiological conditions, maintaining a stable impedance of less than 1 MΩ (at 1 kHz) for the first 30 days to eliminate the need for removal surgery. In vivo, the PPU-based sensor reliably recorded SEPs with amplitudes up to 1196 μV under a 30 μA stimulus. Furthermore, it successfully mapped the spatiotemporal propagation of 4-aminopyridine-induced seizures, accurately capturing both broadband epileptiform discharges and high-frequency oscillations (HFOs).

Expanding the capabilities of PU substrates beyond cortical applications and pushing their mechanical limits, Dong et al. [47] developed a high-resolution (~30 μm) microfluidic electrode array utilizing a thermoplastic polyurethane (TPU) substrate and liquid metal, which withstands over 600% tensile strain and maintains stable impedance (~300 kΩ at 1 kHz) for over 8 weeks in vivo (Figure 4d). When applied to the cerebral cortex, the highly conformable TPU device provided high-fidelity, real-time mapping of pilocarpine-induced epileptiform LFPs. Furthermore, utilizing its internal stress, the polyurethane substrate can self-roll into a tubular cuff (with a minimum inner diameter of 160 μm) to seamlessly wrap around dynamic peripheral nerves with minimal constraint. In vivo, these self-rolled electrodes successfully recorded evoked compound nerve action potentials from the sciatic nerve (0.6–1.0 mV peaks under 50–150 μA stimulation) and vagus nerve (1.5 mV amplitude). For closed-loop neuromodulation, vagus nerve stimulation (VNS) via this flexible interface effectively reduced the heart rate of anesthetized rats (from 285 to 210 bpm) and rabbits (from 250 to 150 bpm). However, these elastomeric materials harbor inherent immunological and toxicological drawbacks that compromise long-term bio-integration. The profound hydrophobicity of PDMS aggressively promotes non-specific protein adsorption (biofouling) immediately upon implantation. This protein layer acts as a potent immunological trigger, aggressively activating macrophages and microglia to initiate a foreign body response. Conversely, while certain polyurethanes offer biodegradability, their in vivo oxidative and hydrolytic degradation can release potentially toxic byproducts, such as diisocyanates or acidic fragments. The localized accumulation of these degradation products can drastically reduce tissue pH, causing severe cytotoxicity and exacerbating chronic neuroinflammation.

Collectively, the integration of intrinsically stretchable elastomers, such as PDMS and PU, has profoundly advanced the development of flexible neural interfaces. By providing tissue-like mechanical compliance, dynamic stress-relaxation, and exceptional elongation, these bulk polymeric substrates effectively mitigate the mechanical mismatch at the tissue–device interface, enabling highly stable chronic recordings and precise neuromodulation across both cortical and peripheral nervous systems [82,83]. However, despite their macroscopic biomechanical advantages, continuous solid elastomeric films inherently lack the micro-porosity and breathability required to fully mimic the natural ECM and promote optimal cellular infiltration. To address these microstructural limitations and further enhance the long-term biocompatibility of brain–computer interfaces, researchers have increasingly turned to electrospun nanofibrous architectures [84]. By offering high surface-area-to-volume ratios, highly tunable interconnected porosity, and superior permeability, electrospun flexible materials represent a compelling evolution in advanced bio-interface design, which will be explored in the following section.

## 5. Electrospun Nanofibrous Microelectrodes

Building directly upon the need to transcend the structural limitations of solid, impermeable elastomers, fibrous membranes, particularly those fabricated via electrospinning, have emerged as a transformative class of flexible substrates for BCIs [85]. Electrospinning is a versatile and highly tunable manufacturing technique that utilizes electrostatic forces to draw charged polymer solutions into continuous, ultrafine fibers, creating non-woven meshes with diameters ranging from nanometers to a few micrometers [86,87]. The resulting 3D architecture profoundly differentiates itself from bulk continuous films by closely mimicking the topological features and nanoscale dimensions of the native ECM [88]. Unlike solid PDMS or PU layers that can physically block interstitial fluid exchange, the highly interconnected porosity and superior permeability of electrospun networks intrinsically facilitate the bidirectional diffusion of oxygen, nutrients, and metabolic waste [89]. This biomimetic microenvironment not only minimizes localized tissue necrosis and chronic inflammatory responses but also actively promotes cellular adhesion, infiltration, and targeted neurite outgrowth. By elegantly combining the macroscopic mechanical compliance of flexible polymers with microscopic biological integration, electrospun substrates establish a critical new paradigm for designing chronically stable and intimately integrated neural electrodes [74].

**Figure 5:**
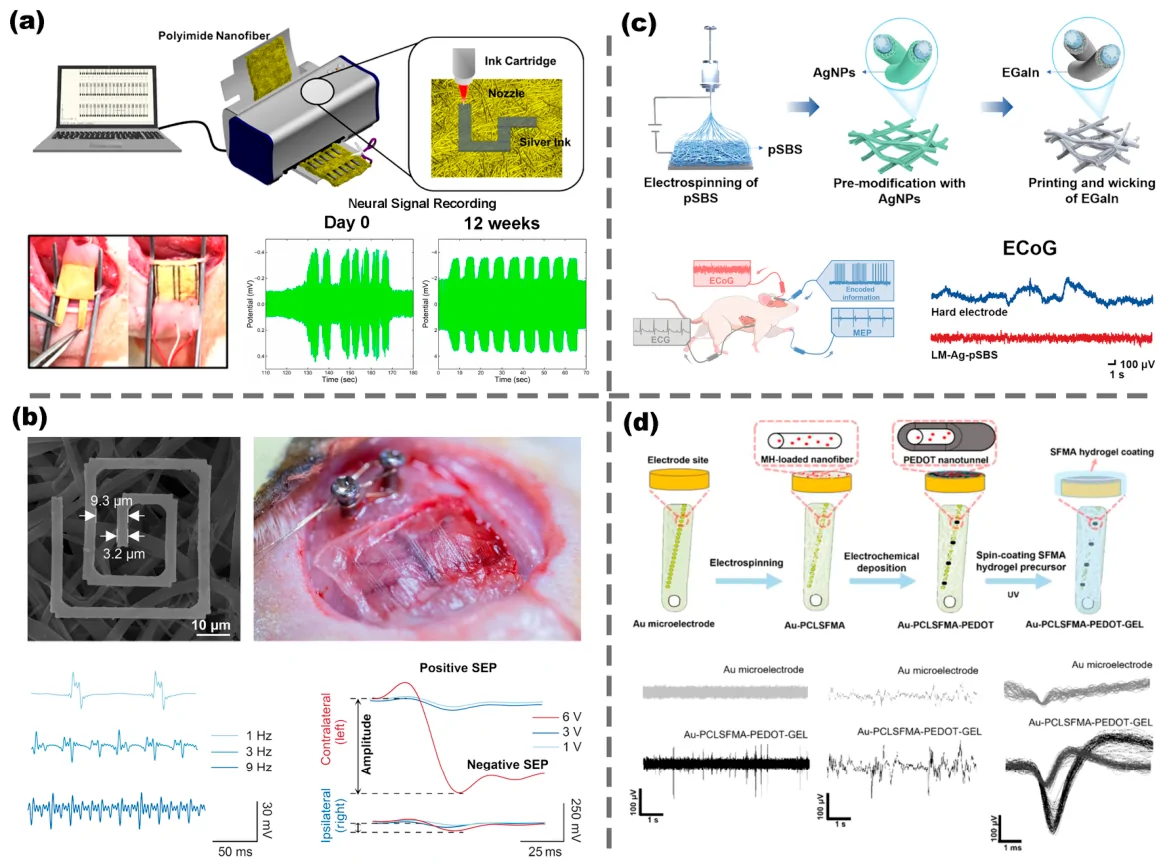
(**a**) Schematic illustration of the fabrication process using an inkjet printing system. Photographs display the electrical packaging and the implanted device. Representative neural signals recorded from sciatic nerve tissue over a 12-week period using a nanofiber (NF)-based electrode loaded with tranilast [48]. (**b**) Scanning electron microscopy image of an electrode on the SBS fiber mat after selective wetting with liquid metal. Digital photograph showing the μLME electrode array conforming to the soft, curved cerebral cortex. SEPs recorded with μLMEs under electrical stimulation using pulse voltages of various frequencies, and a comparison of SEPs in the contralateral and ipsilateral somatosensory cortices under varied stimulation intensities [49]. (**c**) Fabrication process of the LM-Ag-pSBS physiological electrode. Schematic diagram illustrating its application as an implantable sensor for monitoring ECG, ECoG, and MEP signals, alongside a comparison of ECoG recordings between a rigid electrode and the flexible LM-Ag-pSBS electrode [50]. (**d**) Schematic detailing the fabrication of the multifunctional composite coating on an Au-PCLSFMA-PEDOT-GEL microelectrode. Representative 5-s recordings demonstrate robust neural activity from the modified device (black) compared to baseline noise from the pristine Au microelectrode (gray). Filtered LFP data highlights the high-amplitude signals acquired by the modified device. Extracted spike waveforms confirm robust single-unit activity for the Au-PCLSFMA-PEDOT-GEL device, whereas the pristine Au microelectrode remains silent [51].

To realize this biomimetic paradigm, researchers initially utilized robust, biocompatible polymers to construct porous fibrous scaffolds. Heo et al. [48] combined electrospinning and inkjet printing to develop an implantable, highly permeable neural electrode based on PI nanofibers (Figure 5a). The choice of PI for such electrospun architectures is directly dictated by its excellent chemical nature. Because the electrospinning process drastically increases the surface-area-to-volume ratio, the scaffold becomes highly exposed to physiological fluids. The robust, highly polarized imide backbone of PI ensures that even as an ultra-fine fibrous mesh, it retains its extreme chemical inertness and acts as an excellent dielectric barrier, preventing the structural swelling or hydrolytic degradation that typically plagues lesser polymers. Overcoming the limitations of solid films, the nanofiber network (74.0% porosity) exhibits great flexibility with a Young’s modulus of 41.0 MPa and an elongation at break of 40.3%. This biomimetic structure facilitates fluid exchange, effectively preventing tissue compression and nerve atrophy. Coated with patterned silver nanoparticles (AgNPs) and PEDOT:PSS, the device achieved a remarkably low impedance of 123.7 Ω at 1 kHz and a high charge delivery capacity of 188.77 mC·cm^−2^. In vivo, the nanofibrous electrode successfully recorded continuous, high-SNR electroneurogram (ENG) signals from the rat sciatic nerve over a 12-week period, and it captured 0.768 mV evoked action potentials in ex vivo tests. Furthermore, histological analysis confirmed that the porous architecture minimized fibrotic encapsulation and preserved healthy axonal density throughout long-term implantation.

While PI nanofibers provide excellent permeability, advancing toward true tissue-level elasticity requires the integration of elastomeric fibers with intrinsically stretchable conductors. Taking this evolutionary step, Zhuang et al. [49] developed highly permeable and stretchable liquid metal microelectrodes (µLMEs) on an electrospun poly(styrene-block-butadiene-block-styrene) (SBS) mat using wafer-scale photolithography. This approach achieved an ultrahigh resolution of 2 μm and a device density of ~75,500 electrodes/cm^−2^ (Figure 5b). The ultrathin (25 μm) porous substrate exhibited high air (235 mm·s^−1^) and moisture (990 g·m^−2^ per day) permeability, minimizing inflammation and ensuring chronic biocompatibility for over 8 months in vivo. Electrically, the µLMEs maintained high conductivity (up to 3.9 × 10^5^ S·cm^−1^) and stable performance under tensile strains exceeding 1000%. When implanted as a neural interface, the device enabled high-spatiotemporal-resolution ECoG mapping in rats. It reliably recorded sleep-state delta and theta waves, as well as SEPs featuring positive (15–107 mV) and negative (44–630 mV) peaks during forelimb stimulation.

Although liquid metals offer exceptional compliance, their inherent high surface tension often makes wetting and securing them within 3D porous skeletons challenging, which can compromise long-term stability. To overcome this limitation and dramatically enhance mechanical robustness, Zhou et al. [50] developed a highly permeable and durable nanofibrous bio-interface by integrating eutectic gallium-indium (EGaIn) liquid metal into an electrospun poly(styrene-block-butadiene-block-styrene) (pSBS) mat (Figure 5c). By employing a spatial wettability tuning strategy with silver nanoparticles (AgNPs, 0.2 g cm^−3^), the liquid metal wicked seamlessly into the 3D pores, achieving a high conductivity of 5.42 × 10^5^ S·m^−1^, >1000% stretchability, and durability over 20,000 stretch cycles at 60% strain. The porous architecture ensured excellent air (10.6 cm·s^−1^) and moisture (≈1.1 g·m^−2^·h^−1^) permeability, supporting normal tissue metabolism and yielding negligible cortical damage after a one-month in vivo implantation. As an implantable neural interface, this flexible electrode successfully recorded high-fidelity ECoG and electrocardiograph (ECG) signals with a high signal-to-noise ratio of 13.43 dB, and accurately detected intensity-dependent motor evoked potentials (MEPs) under 15 mA and 20 mA stimulations.

Beyond optimizing the physical and electrical properties of the conductive network, electrospun scaffolds can also be engineered to actively manage the biological response at the tissue interface. To expand the utility of electrospun scaffolds beyond simple physical compliance and porosity, Pang et al. [51] utilized electrospinning to construct a multifunctional composite coating that simultaneously addresses high electrochemical impedance, acute inflammation, and chronic glial scarring (Figure 5d). The researchers electrospun a polycaprolactone/silk fibroin-methacrylate (PCL-SFMA) nanofiber mesh loaded with the anti-inflammatory drug minocycline hydrochloride onto a flexible gold microelectrode. This nanofibrous layer served a dual purpose: acting as an initial drug delivery vehicle to mitigate acute implantation trauma, and functioning as a structural template for the subsequent electrodeposition of a highly porous PEDOT conductive network. Encapsulated by a final soft, bioactive SFMA hydrogel layer to minimize chronic mechanical mismatch, this hierarchical interface dramatically increased the electroactive surface area. The modified microelectrode achieved a remarkably high cathodic charge storage capacity of 207.8 ± 3.5 mC·cm^−2^ and a significantly reduced impedance of 38.6 ± 2.6 kΩ at 1 kHz. When implanted into the mouse hippocampus, the device elicited a minimal inflammatory response and successfully captured high-fidelity single-unit spike signals with amplitudes reaching up to 165 μV. Demonstrating excellent long-term stability, the composite-coated microelectrode reliably recorded these neural action potentials, distinguishing clearly between anesthetized and awake states, while maintaining a high average SNR of approximately 20 over an 8-month period in freely moving mice. While electrospun networks successfully mimic the physical ECM and reduce mechanically driven immunity, the integration of advanced conductive nanomaterials introduces novel toxicological paradigms. For instance, the long-term physiological stability of metal-based conductors, such as eutectic gallium-indium (EGaIn) and AgNPs, remains a critical concern. In aggressive biological environments, these materials risk continuous dissolution and the leaching of heavy metal ions (Ag^+^, Ga^3+^, In^3+^) into the cerebral cortex, which could cause cumulative neurotoxicity, oxidative stress, and neuronal apoptosis. Similarly, if carbon-based nanomaterials like CNTs or graphene are improperly anchored and escape the fibrous matrix, they can trigger severe immunological rejection and persistent foreign-body giant cell responses.

In summary, electrospun nanofibrous membranes represent a transformative leap in the design of flexible neural interfaces. By providing a biomimetic 3D architecture, unparalleled permeability, and highly tunable electromechanical properties, these porous substrates effectively overcome the severe limitations of traditional solid elastomers, drastically mitigating foreign-body responses and ensuring seamless, long-term bio-integration. However, despite these profound theoretical and demonstrated advantages, the application of electrospun fibrous materials specifically as primary substrates for implantable bioelectrodes is still in its nascent stages, with relatively few comprehensive studies reported in the literature to date. Given their vast, untapped potential to seamlessly harmonize mechanical compliance, interstitial fluid exchange, and active biochemical modulation, this structural approach stands as a highly promising frontier. Consequently, researchers are strongly encouraged to direct greater attention toward this underexplored domain, as fully leveraging electrospun flexible substrates could fundamentally accelerate the development of next-generation, chronically stable brain–computer interfaces.

## 6. Conclusions and Perspectives

The development of implantable neural microelectrodes has witnessed a remarkable materials evolution, fundamentally driven by the imperative to resolve the profound biomechanical and biological mismatch between synthetic electronic devices and soft neural tissue. The historical transition from rigid inorganic substrates (e.g., silicon) to flexible polymers (e.g., PI, SU-8) enabled out-of-plane conformal contact and deep-tissue penetration without brittle fracture. Subsequently, the shift toward stretchable elastomers (e.g., PDMS, PU) successfully addressed in-plane mechanical compliance, actively absorbing the micro-shear forces generated by continuous physiological pulsations.

However, achieving true, long-term bio-integration requires transcending macroscopic mechanical softness to replicate the complex microenvironment of the brain. Electrospun nanofibrous microelectrodes represent a critical breakthrough in this continuum. By seamlessly mimicking the topological features of the native ECM and providing unparalleled interconnected porosity, these breathable scaffolds effectively eliminate the metabolic blockage and severe FBR inherent to solid polymeric films. Integrating these biomimetic architectures with advanced conductive networks, ranging from nanostructured conductive polymers to highly compliant liquid metals, has yielded unprecedented chronic stability, enabling high-fidelity signal acquisition spanning several months in vivo.

Despite these profound theoretical and demonstrated advantages, the deployment of electrospun fibrous materials as primary substrates for clinical BCIs remains in its nascent stages. To fully unlock the transformative potential of this structural paradigm, future research must direct focused attention toward several key frontiers:

- High-Density Integration and Scalable Manufacturing: Patterning ultra-high-resolution microelectrodes on rough, highly porous nanofibrous substrates remains technologically demanding. Future efforts must refine advanced microfabrication techniques, such as modified wafer-scale photolithography and localized liquid metal templating, to achieve the massive channel densities required for complex neural decoding and closed-loop electrotherapy.
- Active Biochemical Modulation and Detoxification: Beyond passive physical compliance, next-generation nanofibrous scaffolds should increasingly incorporate smart, stimuli-responsive elements. Synergizing electrospun networks with functional hydrogels, targeted anti-inflammatory drug-eluting systems, or neurotrophic factors will shift the interface from merely “evading the immune system” to actively fostering neural regeneration. Furthermore, advanced surface modifications must be developed to definitively seal nanomaterials, preventing the toxicological leaching of metal ions and mitigating protein biofouling to ensure true long-term immunological tolerance.
- Translation to Large Animal Models: While current literature demonstrates remarkable chronic stability and SNR in rodent models, the biomechanical forces and immune responses in larger brains are significantly more complex. Validating the long-term electrochemical durability and mechanical robustness of these highly permeable interfaces in non-human primates is a mandatory milestone preceding human clinical translation.
- Fully Untethered, Multimodal Systems: As these ultra-flexible substrates conform perfectly to dynamic biological tissues, pairing them with wireless power and data transmission modules will enable fully implantable, untethered BCIs. This integration is vital for continuous, naturalistic physiological monitoring without the tethering artifacts that disrupt behavioral studies.

Ultimately, the convergence of nanofibrous bio-interfaces, sophisticated microelectronics, and active tissue engineering heralds a new era for neuroprosthetics. By shifting the overarching design paradigm from simply “minimizing mechanical harm” to “actively mimicking and integrating with biology”, electrospun flexible substrates are poised to fundamentally accelerate the realization of chronically stable, high-performance brain–computer interfaces.

## Figures and Tables

**Table 1:** Chronological evolution and comparative analysis of substrate materials for Brain–Computer Interfaces (BCIs).

Material Paradigm	Representative Materials	Typical Young’s Modulus	Key Advantages	Primary Limitations
Rigid Inorganic	Silicon (e.g., Utah/Michigan arrays) [37,38,39]	~150 GPa	High microfabrication precision; seamless tissue penetration; excellent acute recording fidelity.	Extreme mechanical mismatch; acts as a “micro-blade”; triggers severe chronic FBR and glial scarring.
Flexible Polymeric	Polyimide (PI), SU-8, Parylene C [40,41,42,43]	Low GPa range (e.g., ~2.5 GPa)	Excellent out-of-plane bendability for conformal surface mapping; standard MEMS compatibility.	Inextensible (lacks in-plane stretchability); susceptible to buckling during insertion; cannot fully adapt to 3D deformations.
Stretchable Elastomeric	Polydimethylsiloxane (PDMS), Polyurethane (PU) [44,45,46,47]	1–30 MPa	Intrinsic tissue-like elasticity; multiaxial stretchability; effectively absorbs physiological micro-shear forces.	Dense, impermeable solid film that restricts bidirectional interstitial fluid exchange; lacks biomimetic ECM topographical cues.
Electrospun Nanofibrous	Electrospun SBS, pSBS, PCL-SFMA [48,49,50,51]	Highly tunable (compliant)	3D interconnected porosity mimics native ECM; exceptional permeability; actively reduces FBR and promotes integration.	Deployment as primary substrates is still nascent; challenges remain in ultra-high-density microelectrode patterning and scaling.

## Data Availability

No new data were created or analyzed in this study. Data sharing is not applicable to this article.
